# Factors affecting the evolution of Chinese elderly depression: a cross-sectional study

**DOI:** 10.1186/s12877-021-02675-z

**Published:** 2022-02-08

**Authors:** Longyan Cui, Ding Ding, Junfeng Chen, Minghui Wang, Fanrong He, Shiyang Yu

**Affiliations:** 1 College of Public Health, Dalian Medical University, Dalian, 116044 China; 2 Dalian Municipal Center of Disease prevention and control, Dalian, 116044 China

**Keywords:** Depression, Chinese elder, CHARLS, Tendency

## Abstract

**Background:**

In the past decades, China’s rapid economic growth has been accompanied by rapid changes in lifestyle and an increasing prevalence of mental disorders. This study explored the changes and factors associated with depression among the elderly population of China from 2011 to 2018.

**Method:**

Data were obtained from the China Health and Retirement Longitudinal Study. The total sample size consists of 21,484 individuals aged ≥60 years, and the sample sizes in 2011, 2013, 2015 and 2018 were 5081, 4853, 5207, 6343, respectively. Depressive symptoms were measured by the 10-item Short-Form developed by the Center for Epidemiological Studies Depression.

**Result:**

We found that the tendency in depression rate of the Chinese elderly from 2011 (36.8%) to 2018 (44.5%). The results showed poor health (OR = 3.553), ADL damage (OR = 2.010), multiple chronic diseases (OR = 1.287), and western (OR = 1.777) are risk factors for depression.

**Conclusion:**

The rate of depression of the elderly people in China has risen dramatically. Therefore, additional steps to prevent, treat and care for the affected population are needed, Mental health prevention and treatment strategies should be incorporated into China’s public health policies in a timely manner to mitigate the serious economic burden caused by the increase of depression.

## Introduction

Depression is defined as a multi-dimensional heterogeneous condition, that has a negative impact on the individual’s physical health, quality of life and psychosocial functioning, and may lead to disability and death [1]. Genetic dispositions, health behaviors, physical exercise, adequate sleep, chronic diseases and social support are related to depression according to considerable research [2–5], but the specific pathogenesis of depression is still being explored [6].

Nowadays, Depression has become the second major risk factor affecting people’s health. Approximately 350 million people worldwide suffer from depression [7]. The global economic burden of diseases caused by depression is one of the top five among all types of diseases [8], accounting for 4% of the global burden of disease [6] and the global economic burden of non-fatal diseases of 10% [9]. The difference in the prevalence of depression is caused by regional differences. In high-income countries, such as the proportion of elderly depression is 6.6% (the United States) [10] and 3.7% (Singapore) [11]. In contrast, the incidence of depression in the elderly in low-and middle-income countries is higher [12]. For example, the prevalence of depression in the elderly over 60 years old is 9% (Africa) [8] and 7.8 to 34.8% (Asia) [13]. The challenge of depression in the elderly in developing countries is even more serious.

China is transitioning from a developing to developed country with a large, aging population, and the situation of depression among in the elderly is particularly acute. In recent years, the detection rate of depression in the elderly has reached 33% of the total population [14]. On average, about $42.67 per capita annual medical expense is associated with treatment of symptoms and depression in China [15]. Mental health status has significant effects on individual medical expenses [16]. There are more than 264 million people over the age of 60 in China in 2020 [17], and by 2050, the elderly population is estimated to increase to 480 million [7]. In this sense, it’s necessary to carry out research on the depression of the elderly in China.

Most of the existing studies on depression in the elderly have explored the current prevalence of depression in the elderly on the basis of cross-sectional research [2, 7, 18]. However, the socio-economic status and disease spectrum have also undergone tremendous changes, which may be inferred the influencing factors and forms of the disease change [19, 20]. Therefore, the main goals of this study are: first, to explore the trend of changes in the rate of depression; second, to explore the effects of influencing factors on depression in the elderly over time; and finally, this research not only provides reference for other developing countries to prevent depression in the elderly, but also can provide advice for countries with severe aging.

## Method

### Data sources and samples

The data of this study were derived from The China Health and Retirement Longitudinal Study (CHARLS), a nationally representative longitudinal survey of persons in China 45 years of age or older. Samples were chosen through multistage probability sampling. Firstly, 150 county-level units were randomly chosen with a probability-proportional-to-size (PPS) sampling technique from a sampling frame containing all county-level units with the exception of Tibet. Secondly, the sample was stratified by region, by living in urban or rural counties, and by county-level GDP. Thirdly, as primary sampling units (PSUs), 3 PSUs within each county-level unit (including cun in rural) or (shequ or juweihui in urban) were selected by using PPS sampling. Finally, the sample from 28 provinces, 150 counties, and 450 communities were identified [21]. This study excluded the following types of respondents: under the age of 60; missing scores for depression; missing health-related conditions, such as ADL scores, and health self-assessments, and the total sample size of the study was 21,484.

### Study variables

#### Outcome variable: depressive symptoms

Depressive symptom was measured through a 10-item Brief Epidemiological Research Center Depression (CES-D) [22]. Existing studies have shown that the scale have highly effective and reliability in Chinese elderly [23, 24]. The Cronbach’s alpha in our research is 0.784 (Table 1). The scale’s score is between 0 and 30 by using the four-point Likert scoring method. In addition, there were two key items of reverse score in this scale. With reference to previous studies, we set a critical value of 10 points or higher as the Cut-off points [25]. Depressive symptoms were divided into (0 = no depressive symptoms; 1 = depressive symptoms).

**Table 1:** The distribution of CSED-10 items

Item	(^−^x ± s)	item-total score correlation	Cronbach’s alpha
I was bothered by things that don’t usually bother me	0.97 ± 1.13	0.560	0.739
I had trouble keeping my mind on what I was doing	0.97 ± 1.13	0.484	0.749
I felt depressed	1.00 ± 1.12	0.641	0.728
I felt everything I did was an effort	1.13 ± 1.21	0.547	0.740
I felt hopeful about the future	1.76 ± 1.25	0.122	0.799
I felt fearful	0.40 ± 0.85	0.438	0.757
My sleep was restless	1.21 ± 1.24	0.374	0.765
I was happy	1.46 ± 1.24	0.323	0.772
I felt lonely	0.66 ± 1.06	0.513	0.746
I could not get “going”	0.47 ± 0.92	0.518	0.748
Total Cronbach’s alpha			0.784

Using the overall sample (*N* = 21,484) to test the reliability and validity of CESD-10. Each item’s score as mean ± SD. The Cronbach’s alpha in our study is 0.784 (> 0.7), which means that the reliability and validity of the questionnaire is relatively high

#### Independent variable

The core view of most contemporary depression theories is that stress can increase the risk of depression by stimulating individual cognition and possible biological processes. Relevant studies have also shown that major stressful life events are powerful factors that make depression [25–27]. For example, ADL, chronic diseases, social activities and night sleep have been demonstrated in previous studies. Therefore, these factors were also considered in our research.

The socio-demographic variables considered in the analysis were age (60–69, 70–79,≥80), gender (male, female), marriage (married or unmarried (divorced, widowed, never married)), residence (urban, rural) and geographical region (East, Middle, West). The socio-economic status was determined as the educational level (elementary school and below, junior high school, high school or above). Individual annual income (RMB) were divided into “≤10,000”, “10,000–20,000” and “≥20,000” [7, 18].

Social activities were represented as 0 = no social activities, 1 = participants who participated in at least one social activity. Nighttime sleep were defined as 1 = < 7 h, 2 = ≥7 h [28]. ADL disability (lack of the ability to carry out daily activities) was described as difficulties related to the following six items: dressing, bathing, eating, going to or getting out of bed, going to the toilet, and abstinence. If they cannot complete one of them independently, they were judged to be disabled (0 was defined as = not disabled, 1 = disabled). Participants first performed a five-level assessment of their self-reported health status (very good, good, fair, poor, and very poor). Based on this report, “very good” or “good” was defined as “good”; “fair”; “poor” or “very bad” was expressed as “poor”. Chronic diseases were divided into two groups:(1 = no disease, 2 = one or more chronic diseases) [7].

### Statistical analysis

All statistical analyses were performed using SPSS25. The data was expressed as a percentage of the classification value. χ2 (chi-square) analysis was used to explore the differences in the prevalence of depressive symptoms; on the basis of univariate analysis, binary logistic regression analysis was used to determine the potential risk factors of depressive symptoms. Data were expressed as OR and 95% CI. The test level was 0.05, and the *p*-value < 0.05 was considered statistically significant.

## Result

### The characteristics of respondents and the detection rate of depression

Overall, more than half of the respondents were women (54.1%); 61.8% were 60–69 years old; 79.2% were married. The majority of the respondents had an education level of elementary school and below (79.9%) and the annual income is below 10,000 yuan (70.9%) (Table 2).

**Table 2:** The basic information (n(%))

Variable	Total (*N* = 21,484)	2011 (*N* = 5081)	2013 (*N* = 4853)	2015 (*N* = 5207)	2018 (*N* = 6343)
Area					
East	6938(32.3)	1636(32.2)	1553(32.0)	1665(32.0)	2084(32.9)
Middle	7301(34.0)	1698(33.4)	1655(34.1)	1804(34.6)	2144(33.8)
Western	7245(33.7)	1747(34.4)	1645(33.9)	1738(33.4)	2115(33.3)
Gender					
Male	9857(45.9)	2353(46.3)	2249(46.3)	2354(45.2)	2901(45.7)
Female	11,627(54.1)	2758(53.7)	2604(53.7)	2853(54.8)	3442(54.3)
Age					
60 ~ 69	13,274(61.8)	3033(59.7)	3052(62.9)	3264(62.7)	3925(61.9)
70 ~ 79	6629(30.9)	1620(31.9)	1459(30.1)	1590(30.5)	1960(30.9)
≥ 80	1581(7.3)	428(8.4)	342(7.0)	353(8.8)	458(7.2)
Marriage					
Married	17,022(79.2)	3947(77.7)	3878(79.9)	4117(79.1)	5080(80.1)
Unmarried	4462(20.8)	1134(22.3)	975(22.1)	1090(20.9)	1263(19.9)
Education					
Illiterate or Primary school	17,172(79.9)	4241(83.4)	3970(81.8)	4172(80.1)	4789(75.5)
Middle school	3322(15.5)	664(13.1)	701(14.4)	8212(15.8)	1136(17.9)
High school or above	990(4.6)	176(3.5)	182(3.8)	214(4.1)	418(6.6)
Residence					
Rural	16,268(37.7)	3898(76.7)	3780(77.9)	3993(76.7)	4597(72.5)
City/town	5216(24.3)	1183(23.3)	1073(22.1)	1214(23.3)	1746(27.5)
Individual income					
< 10,000	15,240(70.9)	4620(91.1)	2307(47.5)	4478(86.0)	3829(60.4)
10,000 ~ 20,000	3410(15.9)	290(5.7)	1700(35.1)	193(3.7)	1227(19.3)
≥ 20,000	2834(13.2)	165(3.2)	846(17.4)	536(10.3)	1287(20.3)
Social activity					
Yes	10,697(49.8)	2361(46.5)	2651(54.6)	2583(50.4)	3102(48.9)
No	10,787(50.2)	2720(53.5)	2202(45.4)	2624(49.6)	3241(51.1)
Nighttime sleep					
< 7 h	12,848(59.8)	2957(58.2)	3020(62.2)	3006(57.7)	3865(60.9)
≥ 7 h	8636(40.2)	2124(41.8)	1833(37.8)	2201(42.3)	2478(39.1)
ADL disability					
Yes	2076(9.3)	538(10.6)	424(8.7)	531(10.2)	583(9.2)
No	19,408(90.7)	4543(89.4)	4429(91.3)	4676(89.8)	5760(90.8)
Self-reported heath					
Good	2716(12.6)	452(8.9)	591(12.2)	652(12.5)	1021(16.1)
General	7774(36.2)	1378(27.1)	1622(33.4)	1711(32.9)	3063(48.3)
Poor	10,994(51.2)	3251(64.0)	2640(54.4)	2844(54.6)	2259(35.6)
Chronic disease					
0	6215(28.9)	1005(19.8)	1134(23.4)	965(18.5)	3111(49.0)
≥ 1	15,269(71.1)	4076(80.2)	3719(76.6)	4242(81.5)	3232(51.0)

The detection rates of depression in 2011, 2013, 2015, and 2018 were (36.8%), (38.7%), (42.7%), (44.5%), respectively. The detection rates of newly-added elderly people with depression in 2013, 2015, and 2018 were (37.7%), (40.8%), (41.1%), respectively. In addition, the rates of depression of the elderly who have been repeatedly investigated in the four waves were (37.4%), (40.8%), (45.0%), (49.8%). There are differences in the detection rate of depression among the elderly (including overall, newly added, and repeated) in different years (*P* < 0.05), and the detection rate of depression shows an upward trend (Table 3).

**Table 3:** The detection rate of depression in the elderly in different years

Depression ^a^	2011	2013	2015	2018	Trend	χ2	*P* value
Total						81.256	< 0.001
Yes	1872(36.8)	1876(38.7)	2224(42.7)	2822(44.5)	↑		
No	3209(63.2)	2977(61.3)	2983(57.3)	3521(55.5)			
Add sample ^b^						4.185	0.041
Yes	–	669(37.7)	837(40.8)	1309(41.1)	↑		
No	–	1104(62.3)	1216(59.2)	1877(58.9)			
Duplicate sample ^c^						45.972	< 0.001
Yes	494(37.4)	538(40.8)	594(45.0)	657(49.8)	↑		
No	826(62.6)	782(59.2)	726(55.0)	663(50.2)			

↑This means that the change in the detection rate of depression in the elderly in the fourth phase of the survey shows an upward trend

^a^ Chi-square test is used to explore the difference in the detection rate of different types of elderly depression in different years

^b^ New samples added during interval years, The newly added number of people in the three phases of the 2013, 2015, and 2018 surveys are: (*N* = 1773); (*N* = 2053); (*N* = 3186), respectively

^c^ The elderly people who have been in these four surveys, (*N* = 1320)

### Analysis of depression in the elderly with different characteristics

The results showed that there were differences in the rate of depression in the elderly with different chronic diseases, ADL status, self-reported health, social activities, sleep time and other characteristics (*P* < 0.05). The rate of depressive symptoms in the elderly in the western region, female, 60–69 years old, elementary school education and below, unmarried, suffering from a variety of chronic diseases, ADL disability, and poor health is even higher. However, it is worth noting that in the chi-square analysis in 2018, it was found that there was no difference in the rate of depressive symptoms among elderly people of different ages (*P* = 0.123) (Table 4).

**Table 4:** Chi-square analysis of depression in the elderly with different characteristics

Variable	2011 (*N* = 1872)	*P* value	2013 (*N* = 1876)	*P* value	2015 (*N* = 2224)	*P* value	2018 (*N* = 2822)	*P* value
Area								
East	461(24.6)	< 0.001	486(25.9)	< 0.001	564(25.4)	< 0.001	750(26.6)	< 0.001
Middle	631(33.7)		622(33.2)		792(35.6)		981(34.8)	
Western	780(41.7)		768(40.9)		868(39.0)		1091(38.6)	
Gender								
Male	713(38.1)	< 0.001	735(39.2)	< 0.001	847(38.1)	< 0.001	1107(39.2)	< 0.001
Female	1159(61.9)		1141(60.8)		1137(61.9)		1715(60.8)	
Age								
60 ~ 69	1154(61.6)	0.023	1233(65.7)	0.004	1455(65.4)	< 0.001	1755(62.2)	**0.123**
70 ~ 79	553(29.5)		515(27.5)		648(29.1)		884(31.3)	
≥ 80	165(8.9)		128(6.8)		121(5.6)		183(6.5)	
Marriage								
Married	1392(74.4)	< 0.001	1452(77.4)	0.001	1690(76.0)	< 0.001	2174(77.0)	< 0.001
Unmarried	480(25.6)		424(22.6)		534(24.0)		648(23.0)	
Education								
Illiterate or Primary school	1672(89.3)	< 0.001	1615(86.1)	< 0.001	1907(85.8)	< 0.001	2311(81.9)	< 0.001
Middle school	168(9.0)		205(10.9)		259(11.6)		392(13.9)	
High school or above	32(1.7)		56(3.0)		58(2.6)		119(4.2)	
Residence								
Rural	1576(84.2)	< 0.001	1587(84.6)	< 0.001	1838(82.6)	< 0.001	2224(78.8)	< 0.001
City/town	296(15.8)		289(15.4)		386(17.4)		598(21.2)	
Individual income								
< 10,000	1759(94.0)	< 0.001	958(51.1)	< 0.001	2024(91.0)	< 0.001	1896(67.2)	< 0.001
10,000 ~ 20,000	88(4.7)		698(37.2)		65(2.9)		555(19.7)	
≥ 20,000	25(1.3)		220(11.7)		135(6.1)		371(13.1)	
Social activity								
Yes	757(40.4)	< 0.001	932(49.7)	< 0.001	1003(45.1)	< 0.001	1278(45.3)	< 0.001
No	1115(59.6)		944(50.3)		1221(54.9)		1554(54.7)	
Nighttime sleep								
< 7 h	1278(68.3)	< 0.001	1370(73.0)	< 0.001	1463(65.8)	< 0.001	1967(69.7)	< 0.001
≥ 7 h	594(31.7)		506(27.0)		761(34.2)		855(30.3)	
ADL disability								
Yes	937(50.1)	< 0.001	246(13.1)	< 0.001	336(15.1)	< 0.001	397(14.1)	< 0.001
No	935(49.9)		1630(86.9)		1888(84.9)		2425(85.9)	
Self-reported heath								
Good	71(3.8)	< 0.001	119(6.3)	< 0.001	142(6.4)	< 0.001	243(8.6)	< 0.001
General	359(19.2)		507(27.1)		581(26.1)		1140(40.4)	
Poor	1442(77.0)		1250(66.6)		1501(67.5)		1439(51.0)	
Chronic disease								
0	281(15.0)	< 0.001	349(18.6)	< 0.001	312(14.0)	< 0.001	1230(43.6)	< 0.001
≥ 1	1591(85.0)		1527(81.4)		1912(86.0)		1592(56.4)	

### Logistic regression model

Binary logistic regression analysis was used to explore the influencing factors of depression in the elderly, and the results of each period section show that there is an association between region, gender, marriage, residence, sleep, social activities, self-reported heath, chronic disease, ADL and depression (Table 5).

**Table 5:** Binary logistic regression analysis of depressive symptoms (OR, 95% CI)

Variable	Model 1^a^ (*N* = 5081)	Model 2^b^ (*N* = 4853)	Model 3^c^ (*N* = 5207)	Model 4^d^ (*N* = 6343)
Area (East)	Ref	Ref	Ref	Ref
Middle	1.391^***^(1.190–1.626)	1.279^**^(1.095–1.494)	1.554^***^(1.341–1.802)	1.414^***^(1.235–1.618)
Western	1.791^***^(1.535–2.089)	1.758^***^(1.507–2.050)	1.779^***^(1.534–2.064)	1.740^***^(1.521–1.990)
Gender (male)	Ref	Ref	Ref	Ref
Female	1.569^***^(1.380–1.783)	1.502^***^(1.320–1.709)	1.512^***^(1.336–1.712)	1.514^***^(1.351–1.696)
Age (60 ~ 69)	Ref	Ref	Ref	
70 ~ 79	0.847^*^(0.737–0.973)	0.801^**^(0.695–0.924)	0.771^***^(0.674–0.881)	–
≥80	0.933(0.737–1.181)	0.965(0.743–1.254)	0.547^***^(0.421–0.711)	–
Marriage (married)	Ref	Ref	Ref	Ref
Unmarried	1.233^**^(1.058–1.439)	1.208^*^(1.027–1.421)	1.440^***^(1.235–1.678)	1.224^**^(1.068–1.403)
Education (Illiterate or Primary school)	Ref	Ref	Ref	Ref
Middle school	0.746^**^(0.605–0.920)	0.846(0.693–1.033)	0.744^**^(0.621–0.891)	0.808^**^(0.691–0.944)
High school or above	0.645^*^(0.422–0.986)	1.190(0.830–1.706)	0.799(0.566–1.128)	0.687^**^(0.536–0.880)
Residence (rural)	Ref	Ref	Ref	Ref
City/town	0.600^***^(0.506–0.712)	0.552^***^(0.445–0.684)	0.646^***^(0.550–0.760)	0.703^***^(0.606–0.816)
Individual income (< 10,000)	Ref	Ref	Ref	Ref
10,000 ~ 20,000	0.856(0.649–1.128)	1.014(0.884–1.164)	0.640**(0.462–0.887)	0.965(0.838–1.111)
≥20,000	0.630^*^(0.399–0.994)	0.889(0.693–1.138)	0.625^***^(0.494–0.792)	0.711^***^(0.595–0.851)
Social activity (no)	Ref	Ref	Ref	Ref
Yes	0.796^***^(0.703–0.901)	0.803^**^(0.708–0.911)	0.795^***^(0.705–0.897)	0.897(0.804–1.002)
Nighttime sleep (< 7 h)	Ref	Ref	Ref	Ref
≥7 h	0.552^***^(0.486–0.626)	0.480^***^(0.421–0.548)	0.581^***^(0.515–0.656)	0.567^***^(0.507–0.635)
ADL disability (no)	Ref	Ref	Ref	Ref
Yes	1.931^***^(1.589–2.345)	2.0149^***^(1.621–2.503)	2.129^***^(1.744–2.601)	1.900^***^(1.561–2.313)
Self-reported heath (good)	Ref	Ref	Ref	Ref
General	1.758^***^(1.314–2.351)	1.660^***^(1.311–2.102)	1.620^***^(1.300–2.018)	1.799^***^(1.521–2.127)
Poor	3.477^***^(2.643–4.574)	2.981^***^(2.382–3.732)	3.338^***^(2.707–4.117)	4.392^***^(3.679–5.243)
Chronic disease (0)	Ref	Ref	Ref	Ref
≥1	1.323^**^(1.123–1.559)	1.374^***^(1.180–1.600)	1.379^***^(1.175–1.618)	1.180^**^(1.056–1.317)

Table 5 shows the binary logistic regression analysis of depression in the elderly for each cross-sectional survey

*P < 0.05; **P < 0.01; ***P < 0.001

^a^ The logistic model of 2011; ^b^ The logistic model of 2013; ^c^ The logistic model of 2015; ^d^ The logistic model of 2018, Age was excluded in the model

Then, time (the year of the survey) was also used as a factor, and its effect in depression in the elderly was discussed after adjusting for variables such as socioeconomic characteristics and health status. The results show that poor health (OR = 3.553), ADL disability (OR = 2.010), respondents in 2018 (OR = 2.213), Western (OR = 1.777), women (OR = 1.521); multiple chronic diseases (OR = 1.287) and (OR = 1.294) are risk factors for depression. Sleeping time at night≥7(OR = 0.549), living in town (OR = 0.635), annual income≥20000yuan (OR = 0.741), and social activities (OR = 0.825) are protective factors for depression in the elderly (Table 6).

**Table 6:** Binary logistics regression analysis of overall depression in the elderly (OR, 95% CI)

Variable	Model 1^a^	Model 2^b^	Model 3^c^	Model 4^d^
Years (2011)	Ref	Ref	Ref	Ref
2013	1.080(0.996–1.172)	1.165^**^(1.065–1.273)	1.213^***^(1.114–1.321)	1.287^***^(1.172–1.413)
2015	1.278^***^(1.181–1.383)	1.329^***^(1.225–1.442)	1.449^***^(1.333–1.575)	1.496^***^(1.374–1.630)
2018	1.374^***^(1.274–1.482)	1.568^***^(1.447–1.700)	1.968^***^(1.810–2.140)	2.213^***^(2.025–2.419)
Area (east)		Ref		Ref
Middle	–	1.537^***^(1.433–1.649)	–	1.416^***^(1.315–1.524)
Western	–	1.943^***^(1.812–2.084)	–	1.777^***^(1.652–1.912)
Gender (male)		Ref		Ref
Formale	–	1.534^***^(1.447–1.627)	–	1.521^***^(1.431–1.618)
Age (60 ~ 69)		Ref		Ref
70 ~ 79	–	0.871^***^(0.817–0.928)	–	0.832^***^(0.779–0.890)
≥ 80	–	0.833**(0.743–0.935)	–	0.798^***^(0.707–0.901)
Marriage (married)		Ref		Ref
Unmarried	–	1.262^***^(1.174–1.356)	–	1.294^***^(1.199–1.396)
Education (Illiterate or Primary school)		Ref		Ref
Middle school	–	0.788^***^(0.722–0.860)	–	0.775^***^(0.708–0.849)
High school or above	–	0.743^***^(0.637–0.868)	–	0.771^**^(0.656–0.907)
Residence (rural)		Ref		Ref
City/town	–	0.643^***^(0.593–0.696)	–	0.635^***^(0.584–0.691)
Individual income (< 10,000)		Ref		Ref
10,000 ~ 20,000	–	0.915^*^(0.841–0.995)	–	0.923(0.845–1.007)
≥ 20,000	–	0.695^***^(0.624–0.774)	–	0.741^***^(0.662–0.829)
Social activity (no)			Ref	Ref
Yes	–	–	0.770^***^(0.727–0.816)	0.825^***^(0.777–0.875)
Nighttime sleep (< 7 h)			Ref	Ref
≥ 7 h	–	–	0.543^***^(0.511–0.576)	0.549^***^(0.517–0.584)
ADL disability (no)			Ref	Ref
Yes	–	–	1.991^***^(1.806–2.194)	2.010^***^(1.817–2.222)
Self-reported heath (good)			Ref	Ref
General	–	–	1.735^***^(1.561–1.929)	1.711^***^(1.537–1.906)
Poor	–	–	3.677^***^(3.315–4.079)	3.553^***^(3.197–3.950)
Chronic disease (0)			Ref	Ref
≥ 1	–	–	1.262^***^(1.179–1.351)	1.287^***^(1.200–1.380)

Binary regression analysis of the total sample of the fourth phase of the survey (*N* = 21,484). The results of the chi-square analysis are provided in the attachment, and the results show that there are differences in the detection of depression among the elderly with different gender, age, marriage, income, education, residence, region, sleep time, social activities, self-rated health, and ADL  (Table 7)

*P < 0.05; **P < 0.01; ***P < 0.001

^a^ The basic model, and only the year is added

^b^ On the basis of Model 1, Adjusted age, region, gender, residence, individual income, education and marriage

^c^ Based on Model 1, adjust self-reported health, nighttime sleep, social activities, and ADL

^d^ On the basis of Model 1, health status and socioeconomic characteristics were included

**Table 7:** Chi-square analysis of factors affecting depression (*N* = 21,484)

Variable	Depression (*N* = 8794)	No depression (*N* = 12,690)	χ2	*P* value
Area			367.934	< 0.001
East	2261(25.7)	4677(36.9)		
Middle	3026(34.4)	4275(33.7)		
Western	3507(39.9)	3738(29.4)		
Gender			310.415	< 0.001
Male	3402(38.7)	6455(50.9)		
Female	5392(61.3)	6235(49.1)		
Age			22.945	< 0.001
60 ~ 69	5597(63.6)	5597(60.5)		
70 ~ 79	2600(29.6)	2600(31.7)		
≥ 80	597(6.8)	597(7.8)		
Marriage			78.831	< 0.001
Married	6708(76.3)	10,314(81.3)		
Unmarried	2086(23.7)	2376(18.7)		
Education			277.119	< 0.001
Illiterate or Primary school	7505(85.3)	9667(76.2)		
Middle school	1024(11.6)	2298(18.1)		
High school or above	265(3.1)	725(5.7)		
Residence			335.538	< 0.001
Rural	7225(82.2)	9043(71.3)		
City/town	1569(17.8)	3647(28.7)		
Individual income			287.474	< 0.001
< 10,000	6637(75.5)	8603(67.8)		
10,000 ~ 20,000	1406(16.0)	2004(15.8)		
≥ 20,000	751(8.5)	2083(16.4)		
Social activity			128.555	< 0.001
Yes	3970(45.1)	6727(53.0)		
No	4824(54.9)	5963(47.0)		
Nighttime sleep			537.118	< 0.001
< 7 h	6078(69.1)	6770(53.3)		
≥ 7 h	2716(30.9)	5920(46.7)		
ADL disability			398.793	< 0.001
Yes	1275(14.5)	801(6.3)		
No	7519(85.5)	11,889(93.7)		
Self-reported heath				
Good	575(6.5)	2141(16.9)	1109.081	< 0.001
General	2587(29.4)	5187(40.9)		
Poor	5632(64.1)	5262(42.2)		
Chronic disease			129.559	< 0.001
0	2172(24.7)	4043(31.9)		
≥ 1	6622(75.3)	8647(68.1)		

## Discussion

The study focused on the trend of changes in the rate of depression among the elderly in China. The results show that the rate of depression in the elderly in China has reached 44.5% (in 2018), which is higher than the results of a similar domestic study [7, 14]. Our research also demonstrated that the rate of depressive symptoms showed an upward trend in both the newly-added elderly interviewed and the elderly who have always been in this survey cohort. This finding indicated that the increase in the detection rate of depression caused by time changes is indeed an issue in China, and steps must be taken to improve the mental health condition of elderly.

Research results show that elderly people with poor health (OR = 3.553), ADL impairment (OR = 2.010) and multiple chronic diseases (OR = 1.287) have a higher risk of depression, which is consistent with previous research conclusions [3, 7, 18]. Chronic diseases will cause the health of the elderly to deteriorate, and long-term medication after illness will increase the economic burden of the disease. In addition, due to the damage of ADL, the majority of the elderly rely on the care of family members, which increases the financial burden of the family, and the elderly will also have psychological burden problems. Moreover, it is inconvenient for them to participate in social activities and this prevents them from being able to talk about their inner emotions and may exacerbate depression [29–31].

Sleep is a very important factor affecting health, which has been found to cause many diseases and even all-cause mortality [32]. Our research also indicate that elderly people who sleep for more than 7 h (OR = 0.549) have a lower risk of depression, which is consistent with the study of Gehrman P [33]. Daytime physical fatigue or mental fatigue caused by poor night sleep quality may disrupt the circadian rhythm or cause hormonal changes. Furthermore, lack of sleep at night may be one of the causes of mental disorders or the sequelae of previous mental disorders, and these factors can cause depression produce [32, 34–36].

There is consensus that SES plays an important role in health [37]. Our research found that elderly people with higher personal annual income (OR = 0.741) have a lower risk of depression. It has been suggested that poor SES may lead to poor access to mental health services, and further affect the diagnosis and treatment of depression. It is difficult for low-income populations to attend to healthcare needs and to be screened for depression symptoms [38]. Divorce (OR = 1.294) was found to be a risk factor for depression, which is consistent with the findings of Zhang Y and Ouyang P [7, 39]. As a special group, the elderly may rely more on social support, especially support from family members [40]. The absence of a spouse not only means that the financial support from the spouse is weakened, but also they cannot share the inner feelings with others, This may increase the possibility of depression in the divorced elderly [7, 41].

Social activity (OR = 0.825) was also confirmed as a meaningful factor for depression. The review of empirical literature by Adams et al. reveals that socializing (e.g., spending time with friends, family, or neighbors) has the strongest effect on well-being in late life [42]. By comparison, infrequent participation in social activities is an indicator of social isolation [43].What’s more, events involving interpersonal stress and social rejection are the strongest risk factors for depressive symptoms [44]. Hence social activities should be promoted as an important means of preventing depression in the elderly.

In addition to the factors discussed above, we also found that the West region (OR = 1.777), women (OR = 1.521), higher education (OR = 0.771), and living in city (OR = 0.635) are associated with depression. Considerable research demonstrates that these factors are very important in terms of the potential harm associated with depression [9, 18, 38, 45]. In areas with better economic conditions, the health-related policies, living surroundings and community infrastructure always show better conditions, which are effective in promoting mental health [45, 46]. Experience with rejection, criticism and separation are the factors leading to depression. Women are more likely to perceive these emotions, which leads to a higher likelihood of them suffering from depression [47].

Besides, we also explored the effect of the passage of time on depression in the elderly. This study confirmed that the rate of depression among the elderly in China has increased, and the rate of depressive symptoms increased by nearly 10% from 2011 (36.8%) to 2018 (44.5%). Compared with studies in similar time periods, for example, 32.6% ~ 41.6% (Hu bei, 2011–2015) [48], 39.86% (Jing Yu, 2012) [2], 46.15% (Fang M, 2015) [18]. Our research are roughly consistent with their research results. In general, the level of depression among the elderly in China is gradually increasing. The reasons for this phenomenon are as follows: Firstly, According to the 6th Chinese Census, the elderly living alone or with their spouse accounted for more than 50% of the total elderly population; this number (the 5th Census was 38%) has risen Nearly 12% in decades [49]. The problem of urbanization and empty nesting accompanied by rapid social and economic development is not conducive to the mental health of the elderly [50]. Secondly, the deterioration of physical function will cause the elderly to suffer from chronic diseases and impaired ability of daily living. The heavy economic burden of the disease and the pressure of daily care will lead to depression. Finally, mental health services for the elderly were added to existing public health policies. In addition, the health administration has also implemented mental care projects for the elderly (including monitoring of diseases such as depression or anxiety, and exploring comprehensive community interventions). However, the effects of mental health interventions for the elderly are affected in many ways, such as the level of regional economy, medical resources (professional institutions, caregivers), and the degree of self-esteem of the elderly. Therefore, it’s urgent to improve the mental health intervention measures for the elderly to avoid other public health problems caused by the deterioration of mental health problems.

### Strengths and limitations

Interpretation of these findings should take into consideration several limitations. Firstly, since this study used cross-sectional analysis, we are unable to draw conclusions about whether there is a clear causal relationship between depressive symptoms and research factors. Secondly, the depression detection scale in this study can only be used as a reference opinion, and the diagnosis requires further clinical diagnosis. Finally, there were limited considerations about the factors that affect the occurrence of depression in the elderly, and subsequent research could further explore the role of depression and other factors under the influence of time.

## Conclusion

These above conclusions shed light on the urgent need for reforming the current mental health system in China, and further government involvement is required to improve the treatment and prevention of the mental health conditions. The following suggestions are put forward for the depression of the elderly:(1) The government should use the power of the social organizations to deal with the various needs of the elderly, improve the quality of life of older people. For example, the establishment of a community mental health care center for the elderly. (2) Make full use of the various resources in the community to promote various elderly care services in the community, and provide paid family services (such as nannies and hourly labor caregivers) for the elderly who are inconvenient or difficult to live with. This would include purchasing daily necessities, sick care, providing company, etc. They might assist the elderly to establish cultural organizations and activities for the elderly according to their own interests, which enriches the spiritual life of the elderly. (3) Family members should play an irreplaceable function of spiritual support for the elderly and pay attention to the changes in the mental health of the elderly in time. As well as advocating the elderly to maintain an optimistic attitude towards life.

## Data Availability

The datasets used and/or analyzed during the current study are available from the corresponding author on reasonable request.

## Abbreviations

SES: Socioeconomic Status
ADL: Activities of Daily Living

## References

[1] Atkins R (2010). Self-efficacy and the promotion of health for depressed single mothers. Ment Health Fam Med.

[2] Yu J, Li J, Cuijpers P, Wu S, Wu Z (2012). Prevalence and correlates of depressive symptoms in Chinese older adults: a population-based study. Int J Geriatr Psychiatry..

[3] Alexopoulos GS (2005). Depression in the elderly. Lancet..

[4] Zhai L, Zhang H, Zhang D (2015). Sleep duration and depression among adults: a meta-analysis of prospective studies. Depress Anxiety..

[5] Tough H, Siegrist J, Fekete C (2017). Social relationships, mental health and wellbeing in physical disability: a systematic review. BMC Public Health.

[6] Gałecki P, Talarowska M (2017). The evolutionary theory of depression. Med Sci Monit.

[7] Zhang Y, Liu Z, Zhang L, Zhu P, Wang X, Huang Y (2019). Association of living arrangements with depressive symptoms among older adults in China: a cross-sectional study. BMC Public Health.

[8] Dadi AF, Wolde HF, Baraki AG, Akalu TY (2020). Epidemiology of antenatal depression in Africa: a systematic review and meta-analysis. BMC Pregnancy Childbirth.

[9] Salk RH, Hyde JS, Abramson LY (2017). Gender differences in depression in representative national samples: Meta-analyses of diagnoses and symptoms. Psychol Bull.

[10] Dallo FJ, Prabhakar D, Ruterbusch J, Schwartz K, Peterson EL, Liu B (2018). Screening and follow-up for depression among Arab Americans. Depress Anxiety.

[11] Lau YW, Vaingankar JA, Abdin E, Shafie S, Jeyagurunathan A, Zhang Y (2019). Social support network typologies and their association with dementia and depression among older adults in Singapore: a cross-sectional analysis. BMJ Open.

[12] Gureje O (2020). Late-life depression in socially and culturally diverse settings. Epidemiol Psychiatr Sci.

[13] Tengku Mohd TAM, Yunus RM, Hairi F, Hairi NN, Choo WY (2019). Social support and depression among community dwelling older adults in Asia: a systematic review. BMJ Open.

[14] Peking University National Development Institute released a research report on the elderly: the elderly smoke less and drink more. https://www.thepaper.cn/newsDetail_forward_1546573 (Accessed 14 Oct 2021).

[15] Hsieh CR, Qin X (2018). Depression hurts, depression costs: the medical spending attributable to depression and depressive symptoms in China. Health Econ.

[16] Sun X, Zhou M, Huang L, Nuse B (2020). Depressive costs: medical expenditures on depression and depressive symptoms among rural elderly in China. Public Health.

[17] Main data of the seventh national census. http://www.stats.gov.cn/tjsj/zxfb/202105/t20210510_1817176.html (Accessed 25 June 2021).

[18] Fang M, Mirutse G, Guo L, Ma X (2019). Role of socioeconomic status and housing conditions in geriatric depression in rural China: a cross-sectional study. BMJ Open.

[19] Ryder AG, Sun J, Zhu X, Yao S, Chentsova-Dutton YE (2012). Depression in China: integrating developmental psychopathology and cultural-clinical psychology. J Clin Child Adolesc Psychol.

[20] Hall CA, Reynolds-Iii CF (2014). Late-life depression in the primary care setting: challenges, collaborative care, and prevention. Maturitas..

[21] Zhao Y, Hu Y, Smith JP, Strauss J, Yang G (2014). Cohort profile: the China health and retirement longitudinal study (CHARLS). Int J Epidemiol.

[22] http://charls.pku.edu.cn/articles/news/579/zh-cn.html. (Accessed 25 June 2021).

[23] Andresen EM, Malmgren JA, Carter WB, Patrick DL (1994). Screening for depression in well older adults: evaluation of a short form of the CES-D (Center for Epidemiologic Studies Depression Scale). Am J Prev Med.

[24] Lee AE, Chokkanathan S (2008). Factor structure of the 10-item CES-D scale among community dwelling older adults in Singapore. Int J Geriatr Psychiatry.

[25] Qingbo H, Xiaohua W, Gong C (2015). Reliability and validity of 10-item CES-D among middle aged and older adults in China. China J Health Psychol.

[26] Cheng HG, Chen S, McBride O, Phillips MR (2016). Prospective relationship of depressive symptoms, drinking, and tobacco smoking among middle-aged and elderly community-dwelling adults: results from the China health and retirement longitudinal study (CHARLS). J Affect Disord.

[27] Slavich GM, Irwin MR (2014). From stress to inflammation and major depressive disorder: a social signal transduction theory of depression. Psychol Bull.

[28] Slavich GM, Sacher J (2019). Stress, sex hormones, inflammation, and major depressive disorder: extending social signal transduction theory of depression to account for sex differences in mood disorders. Psychopharmacology.

[29] Colodro-Conde L, Couvy-Duchesne B, Zhu G, Coventry WL, Byrne EM, Gordon S (2018). A direct test of the diathesis-stress model for depression. Mol Psychiatry.

[30] Whinnery J, Jackson N, Rattanaumpawan P, Grandner MA (2014). Short and long sleep duration associated with race/ethnicity, sociodemographics, and socioeconomic position. Sleep..

[31] Li LW, Liu J, Xu H, Zhang Z (2016). Understanding rural-urban differences in depressive symptoms among older adults in China. J Aging Health.

[32] Hong J, Aspey L, Bao G, Haynes T, Lim SS, Drenkard C (2019). Chronic cutaneous lupus erythematosus: depression burden and associated factors. Am J Clin Dermatol.

[33] Patten SB, Williams JVA, Lavorato DH, Wang JL, Jetté N, Sajobi TT (2018). Patterns of association of chronic medical conditions and major depression. Epidemiol Psychiatr Sci..

[34] Li Y, Wu Y, Zhai L, Wang T, Sun Y, Zhang D. Longitudinal association of sleep duration with depressive symptoms among middle-aged and older Chinese. Sci Rep. 2017;7(1). 10.1038/s41598-017-12182-0. 10.1038/s41598-017-12182-0 PMC5603546 28924238

[35] Gehrman P, Seelig AD, Jacobson IG, Boyko EJ, Hooper TI, Gackstetter GD (2013). Predeployment sleep duration and insomnia symptoms as risk factors for new-onset mental health disorders following military deployment. Sleep..

[36] Luik AI, Zuurbier LA, Direk N, Hofman A, Van Someren EJ, Tiemeier H (2015). 24-hour activity rhythm and sleep disturbances in depression and anxiety: a population-based study of middle-aged and older persons. Depress Anxiety..

[37] Shen J, Barbera J, Shapiro CM (2006). Distinguishing sleepiness and fatigue: focus on definition and measurement. Sleep Med Rev.

[38] van Noorden MS, van Fenema EM, van der Wee NJ, Zitman FG, Giltay EJ (2012). Predicting outcome of depression using the depressive symptom profile: the Leiden routine outcome monitoring study. Depress Anxiety..

[39] Wang J, Geng L. Effects of socioeconomic status on physical and psychological health: lifestyle as a mediator. Int J Environ Res Public Health. 2019;16(2). 10.3390/ijerph16020281. 10.3390/ijerph16020281 PMC6352250 30669511

[40] Ng CW, Tan WS, Gunapal PP, Wong LY, Heng BH (2014). Association of Socioeconomic Status (SES) and social support with depressive symptoms among the elderly in Singapore. Ann Acad Med Singap.

[41] Ouyang P, Sun W (2018). The association between depressive symptoms and fall accidents among middle-aged and elderly people in China. Environ Health Prev Med.

[42] Chan A, Malhotra C, Malhotra R, Ostbye T (2011). Living arrangements, social networks and depressive symptoms among older men and women in Singapore. Int J Geriatr Psychiatry..

[43] Kooshiar H, Yahaya N, Hamid TA, Abu Samah A, Sedaghat JV (2012). Living arrangement and life satisfaction in older Malaysians: the mediating role of social support function. PLoS One.

[44] Holtfreter K, Reisig MD, Turanovic JJ (2017). Depression and infrequent participation in social activities among older adults: the moderating role of high-quality familial ties. Aging Ment Health.

[45] Sander R (2005). Preventing social isolation and loneliness among older people: a systematic review of health promotion interventions. Nurs Older People.

[46] Kendler KS, Hettema JM, Butera F, Gardner CO, Prescott CA (2003). Life event dimensions of loss, humiliation, entrapment, and danger in the prediction of onsets of major depression and generalized anxiety. Arch Gen Psychiatry.

[47] Lorant V, Deliège D, Eaton W, Robert A, Philippot P, Ansseau M (2003). Socioeconomic inequalities in depression: a meta-analysis. Am J Epidemiol.

[48] Hu Y, Li B (2021). Temporal trend of prevalence of depressive symptoms and associated factors among Chinese older adults:an analysis based on the CHARLS panel data. Chin Gen Pract.

[49] Sun J (2013). Current situation and changing patterns of living arrangement of Chinese elderly:an analysis based on data from the fifth and sixth censuses of China. Popul Res.

[50] Fang EF, Scheibye-Knudsen M, Jahn HJ, Li J, Ling L, Guo H (2015). A research agenda for aging in China in the 21st century. Ageing Res Rev.

